# How Medicines Act

**Published:** 1892-06

**Authors:** 


### How Medicines Act.

Medicines act, when given internally, by being transformed in nature's human laboratory. In this breaking up into ultimate elements kinetic energy is evolved from the potential energy previously locked up in the drug. This released force acts upon the structures of the body, bringing about certain changes in vital operations. It is these changes which favorably or unfavorably influence morbid processes.

